# Effect of liquid cooling on PCR performance with the parametric study of cross-section shapes of microchannels

**DOI:** 10.1038/s41598-021-95446-0

**Published:** 2021-08-09

**Authors:** Yousef Alihosseini, Mohammad Reza Azaddel, Sahel Moslemi, Mehdi Mohammadi, Ali Pormohammad, Mohammad Zabetian Targhi, Mohammad Mahdi Heyhat

**Affiliations:** 1 Faculty of Mechanical Engineering, Tarbiat Modares University, Tehran, Iran; 2 Faculty of Chemical Engineering, Sahand University of Technology, Tabriz, Iran; 3 Department of Mechanical and Manufacturing Engineering, University of Calgary, Calgary, AB T2N 1N4 Canada; 4 Biological Science Department, University of Calgary, Calgary, AB T2N 1N4 Canada

**Keywords:** Energy science and technology, Mechanical engineering

## Abstract

In recent years, PCR-based methods as a rapid and high accurate technique in the industry and medical fields have been expanded rapidly. Where we are faced with the COVID-19 pandemic, the necessity of a rapid diagnosis has felt more than ever. In the current interdisciplinary study, we have proposed, developed, and characterized a state-of-the-art liquid cooling design to accelerate the PCR procedure. A numerical simulation approach is utilized to evaluate 15 different cross-sections of the microchannel heat sink and select the best shape to achieve this goal. Also, crucial heat sink parameters are characterized, e.g., heat transfer coefficient, pressure drop, performance evaluation criteria, and fluid flow. The achieved result showed that the circular cross-section is the most efficient shape for the microchannel heat sink, which has a maximum heat transfer enhancement of 25% compared to the square shape at the Reynolds number of 1150. In the next phase of the study, the circular cross-section microchannel is located below the PCR device to evaluate the cooling rate of the PCR. Also, the results demonstrate that it takes 16.5 s to cool saliva samples in the PCR well, which saves up to 157.5 s for the whole amplification procedure compared to the conventional air fans. Another advantage of using the microchannel heat sink is that it takes up a little space compared to other common cooling methods.

## Introduction

Nowadays, most of the diagnoses are laboratory-based, and molecular methods are one of the rapid and accurate techniques in this regard. A wide variety of molecular methods are being used for different approaches; PCR is one of the well-known and widely used molecular techniques^1^. Synthesis of several copy numbers of a short length of oligonucleotide (single-stranded DNA) from a template DNA by DNA polymerases was the original concept for PCR, initially introduced by Kary Mullis in 1983^2,3^. This method has very high accuracy due to targeting particular sequences of a templet DNA. Furthermore, because just a few template DNA is good enough for detection by this method, it has very low limit detection potency compared to other detection methods such as ELISA. Therefore, the application of PCR for different clinical and industrial approaches has started to grow remarkably^4,5^.

Due to the stability and availability of air as a coolant, fans and fins were commonly used in traditional electronic devices to cool microchips or microtubes^6,7^. With the advancement of the technology, the generated heat was increased; inconsequent, the thermal conductivity of air inhibits heat to completely remove (not more than 10^6^ W/m^2^) from devices^8,9^. Therefore, liquid cooling is a much more efficient way to enhance PCR performance rather than air cooling. Various parameters affect the performance of the cooling process, such as the pattern of microchannels^10,11^, manifold of inlets/outlets, and shape of cross-sections as geometrical parameters, as well as multiphase fluids such as nanofluids^12^, boiling^13^, and phase change materials (PCMs)^14^. Zhao et al. studied the effect of nanofluids with 0.0%, 0.1%, 0.2%, 0.3%, 0.4%, 0.5% mass fractions on the efficiency of CPU cooling, and their results highlighted that nanofluids with the mass fraction of 0.3% and 0.4% have the best cooling performance in rectangular grooves structure and cylindrical bulges structure respectively^15^. In the other study of Zhao et al., which was investigated experimentally, the decrement of thermal efficiency with mass fraction in relatively lower Reynolds numbers was observed^16^. Also, other techniques increase the efficiency of microchips cooling, such as magnetic field effects on thermo-hydraulic behaviors of magnetic nanofluids^17^. The cross-sections of microchannels cause the heat transfer rate is changed with changes in the area wetted, which some studies are mentioned below.

Deng et al.^18^ investigated five different cross-sections of triangular, rectangular, circular, trapezoidal, and reentrant-shape with an average hydraulic diameter of 0.8 mm for all of the shapes. A uniform heat flux of 12.67 W/cm^2^ is applied on the bottom surface as a thermal boundary condition, and their results demonstrated that the rectangle and trapezoidal cross-sections were associated with minimum and maximum thermal resistance, respectively. In other words, the thermal resistance of the rectangle had an approximately 40% improvement among all cases. Besides, the highest and lowest pressure drop of cases belongs to trapezoidal and triangle, respectively. Wang et al. and Gunnasegaran et al.^19,20^ studied rectangular, triangular, and trapezoidal cross-section shapes. Both studies demonstrated that the rectangular shape is more effective in heat transfer than in other cases. Furthermore, the rectangular shape had the lowest pressure drop compared with other cross-sections.

Also, Tullius et al.^21^ studied square, triangular, circular, hexagonal, diamond, and ellipse cross-section of microchannels under 1 − 15 × 10^5^ W/m^2^ of supplied heat flux, and they reported that the circular cross-section presented the highest efficiency compared to other cross-sections. Fin shapes had Nu values within 37% of each other at high Re, with the triangle fins as the highest and the circle and ellipse fins lower. Alfaryjat et al.^22^ analyzed the thermal–hydraulic for non-conventional cross-section, including rhombus, circular, and hexagonal shapes applying 500 kW/m^2^ on the top plate and Reynolds number from 100 to 1000. They demonstrated that both the heat transfer coefficient and pressure drop increased with the Reynolds number's increment for all cross-sections. Also, the hexagonal cross-section owns the highest heat transfer coefficient (8.2% more than rhombus and 3.75% more than circular). However, it brings the penalty of the highest pressure drop compared to other cases.

Furthermore, Salimpour et al.^23^ reported that the square cross-section microchannel presented a maximum amount of heat transfer (per unit volume of the channel) compared to circular, square, and isosceles right triangle cross-sections. Moreover, Alihosseini et al.^24,25^ reviewed the critical parameters to enhance the heat transfer in a microchannel, e.g., the evaluation criteria index (PEC or η), which is vital in evaluating heat transfer and pressure drop. The results illustrated that the heat transfer enhancement of rectangular microchannel cross-section prevailed the pressure drop penalty. According to the literature review, a comprehensive study about the effect of the cross-section of microchannels is felt for investigation. Also, the liquid cooling effect on the PCR devices could be an appropriate subject that has not been performed before.

In the current study, the effect of applying liquid cooling in the PCR apparatus versus traditional air cooling is compared and investigated numerically. The required time for each cycle is a significant parameter for the improvement of PCR efficiency. A small fan is utilized in a conventional PCR device for the cooling process, which is time-consuming. For solving this problem, the liquid-based cooling approach is introduced and analyzed in the current study. This method enhances the heat transfer rate from samples to the cooling fluid compared to the air-cooling method due to the remarkable higher surface area to volume ratio of a microchannel. This state-of-the-art method leads to reduces the PCR cycle time significantly. Many parameters, i.e., the Nusselt number, pressure drop, and fluid flow pattern in cross-sections, are analyzed in detail. Also, in previous researches, the gap of comparing and balancing between heat transfer and pressure drop is observed. Therefore, the performance evaluation criteria index is calculated and compared for all shapes at different Reynolds numbers. By applying this method for cooling PCR devices, the duration of the process can be reduced significantly, leading to a faster diagnosis for all the PCR applications.

## The characteristics of the geometric shape of microchannel

**Figure 1:**
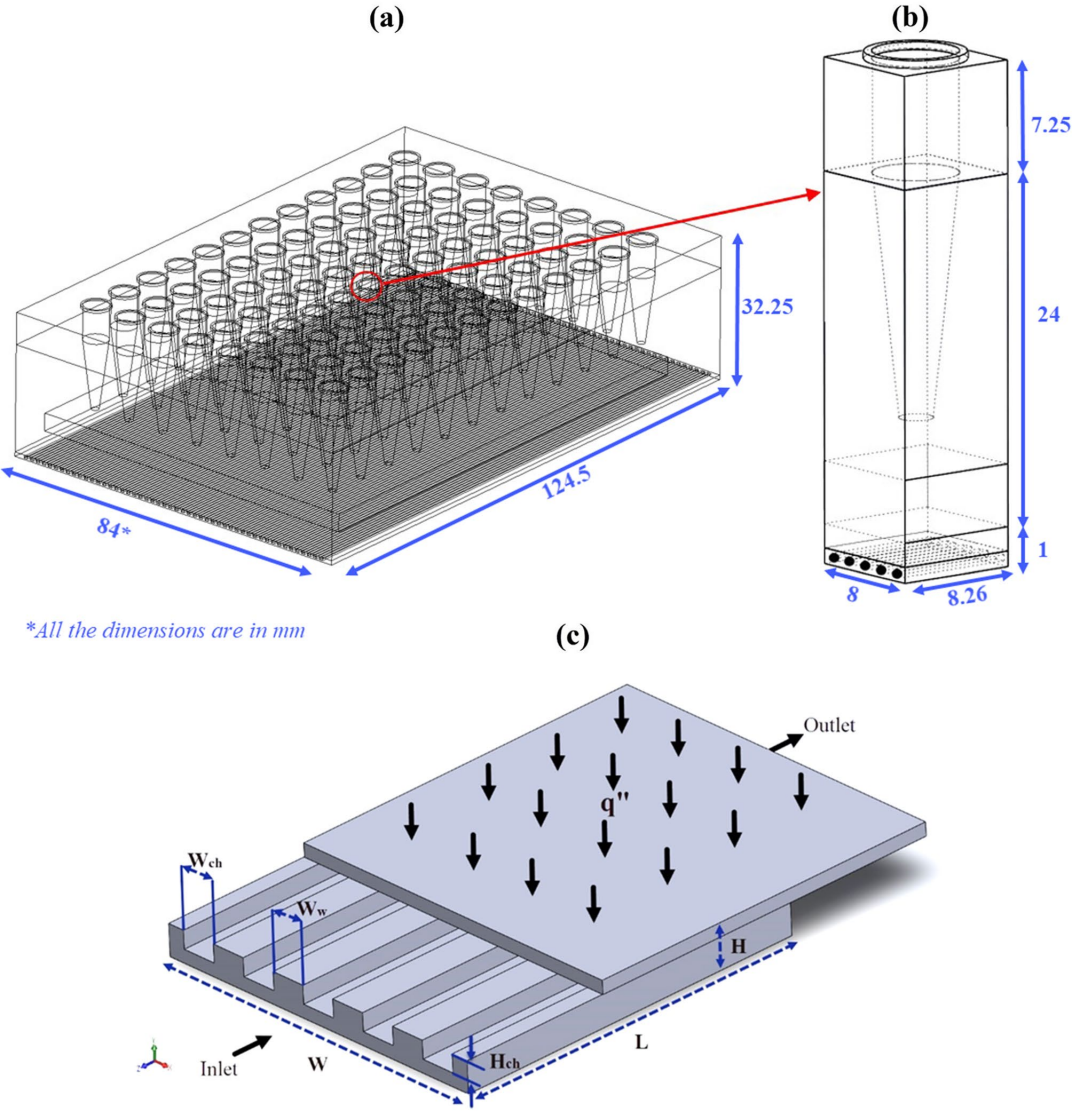
Schematic of (**a**) whole structure of 96 well PCR plate with a microchannel attached to the bottom of the apparatus (**b**) single structure of PCR device and PCR plate with a microchannel attached to the bottom surface, (**c**) microchannel heatsink.

The microchannel schematic diagram is shown in Fig. 1, including labels, detached upper surface, channels, and walls. The heat sink's size is 8 × 8.26 × 1 mm (W, L, and H, respectively), and copper and water are chosen as microchannel material and coolant, respectively; see material properties in Table 1. Also, fifteen different geometry designs, including almost all the typical microchannel cross-sections, are considered to study the impact of cross-section shapes on heat transfer and pressure drop at different Reynolds numbers are presented in Table 2. For all cross-sections, the constant hydraulic diameter and the wall width are adjusted to 533 µm and 800 µm, respectively, based on Deng et al.^18^. Moreover, the fluid flow patterns on each cross-section are investigated in the current paper. Five microchannel cross-sections were chosen as the primary domain among 50 microchannels and then simulated. Heat flux of 103,057 W/m^2^ was applied on the top surface with an isolated bottom surface and sidewalls.

**Table 1:** Thermophysical properties of materials^26^.

Material	Density (kg/m^3^)	Specific heat capacity (J/kg K)	Thermal conductivity coefficient (W/m K)	Cinematic viscosity (kg/m s)
Water	997	4179	0.613	8.55 × 10^−4^
Copper	8933	385	401	–
Air	1.225	1006.43	0.0242	1.78 × 10^−5^

**Table 2:**
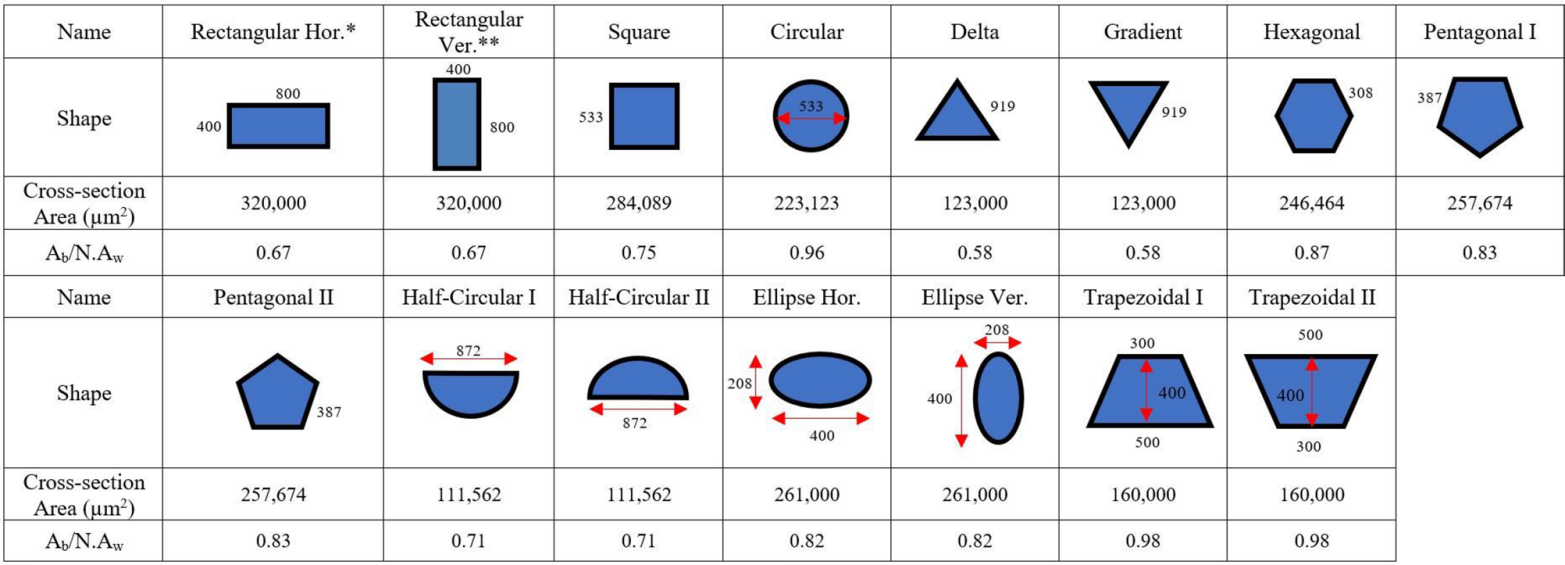
Different microchannel cross-sections with hydraulic diameters of 533 µm.

*Hor., Horizontal.

**Ver., Vertical (all dimensions are in microns).

## Numerical simulation

### Governing equations

A few assumptions are considered to simplify our models for simulation (1) A single-phase with steady-state flow condition, laminar and incompressible, (2) Neglected effect of heat dissipation and gravitational force was caused by viscosity, (3) Natural convection and radiation heat transfer were also neglected. According to assumptions made above, the governing equations are stated as follows:

Mass conservation equation^27,28^:

1 $${\varvec{\rho}}\nabla .\left( {\vec{\user2{U}}} \right) = 0$$

Momentum equation:

2 $$\rho_{f} \frac{{\partial \vec{U}}}{\partial t} + \rho_{f} \left( {\vec{U}.\nabla } \right)\vec{U} = - \nabla p + \mu \nabla^{2} \vec{U}$$

The energy equation for the liquid:

3 $$\rho_{f} C_{{p_{f} }} \frac{\partial T}{{\partial t}} + \rho_{f} C_{{p_{f} }} \left( {\vec{U}.\nabla T} \right) = k_{f} \nabla^{2} T$$

The energy equation for the solid body:

4 $$\rho_{s} C_{{P_{s} }} \frac{\partial T}{{\partial t}} = k_{s} \mathop \nabla \nolimits^{2} T$$

The local bulk temperature of the fluid is measured by Eq. (5):

5 $$T_{f,x} = \frac{{\mathop \smallint \nolimits_{{A_{c} }}^{{}} \rho uC_{p} T dA_{c} }}{{\mathop \smallint \nolimits_{{A_{c} }}^{{}} \rho uC_{p} dA_{c} }}$$

The Average local temperature of the microchannel wall is expressed by Eq. (6):

6 $$T_{\omega .x} = \frac{1}{W}\mathop \smallint \limits_{W} T_{W} dW$$

Then, the Local heat transfer coefficient is calculated by the Eq. (7):

7 $$h_{x} = \frac{{q^{\prime\prime} A_{b} }}{{\left( {T_{W,x} - T_{f,x} } \right) N A_{i} }}$$

The average Nusselt number of the microchannel is evaluated by the Eqs. (8) and (9):

8 $$Nu_{x} = \frac{{h_{x} D_{h} }}{{k_{f} }}$$

9 $$Nu = \frac{1}{L}\mathop \smallint \limits_{L} Nu_{x} dx$$

The pressure drop is measured by the Eq. (10):

10 $$\Delta P = Pout{-}Pin$$

Also, to normalize the geometry dimensions, the microchannel length is divided into ten parts and is assigned as x′.

### Boundary conditions

**Figure 2:**
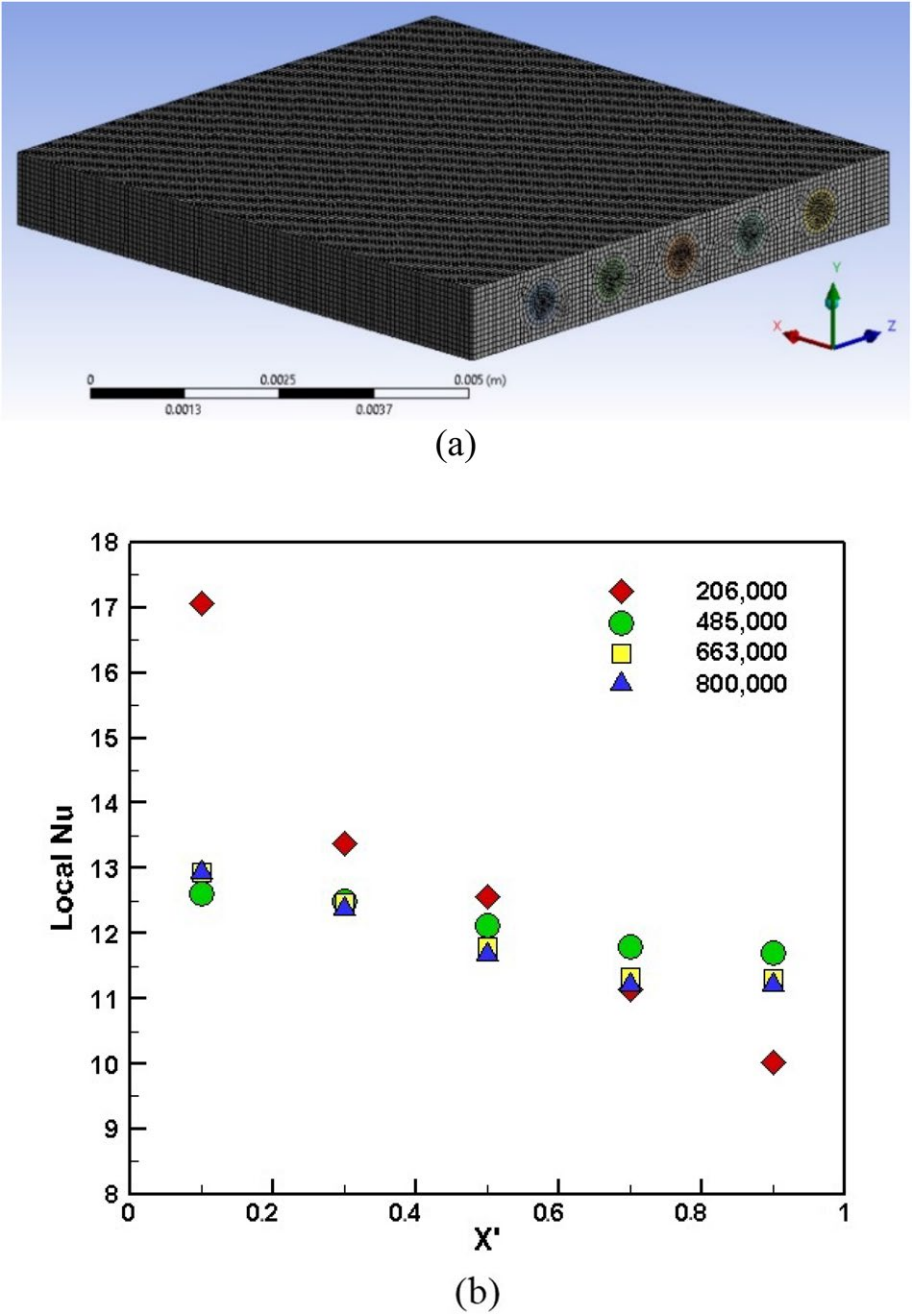
(**a**) 3D model with mesh in ANSYS meshing VER.18.2, (**b**) Mesh independency of local Nusselt number versus the normalized location of microchannel.

The heat transfer and pressure drop were simulated in 3D models using ANSYS Fluent version 18.2 (see Fig. 2a. Also, the heat flux of 103,057 W/m^2^ was applied to the substrate's top surface. The velocity and temperature of the liquid were considered uniform at the microchannel inlet. Moreover, the atmospheric pressure was applied on the outlet boundary condition, and the no-slip boundary condition on the internal walls (fluid velocity was specified zero) was considered. The following equations also demonstrate the boundary conditions explained above in Table 3.

**Table 3:** Boundary conditions of the microchannel heat sink.

Inlet	Outlet	Bottom	Top	Interface	Sides
$u = u_{in}$	*P* = *P*_*out*_	$q^{\prime \prime } = - k_{s} \frac{{\partial T_{s} }}{\partial n} = 0$	$q^{\prime \prime } = - k_{s} \frac{{\partial T_{s} }}{\partial n} = const$	$u = 0$	$q^{\prime \prime } = - k_{s} \frac{{\partial T_{s} }}{\partial n} = 0$
$v = 0$				$v = 0$	
$w = 0$				$w = 0$	
$T = T_{in}$				$T_{s} = T_{f}$	
				$$k_{s} \frac{{\partial T_{s} }}{\partial n} =_{{}} k_{f} \frac{{\partial T_{f} }}{\partial n}$$	

### Mesh independency

Mesh independency was investigated for different element numbers, 206,000, 485,000, 663,000, and 800,000 using a tetrahedral mesh structure. The Nusselt number was simulated for each mesh structure to evaluate the performance of the heat sinks at the Reynolds number of 500, see Fig. 2b. The achieved results demonstrated that an increasing number of elements leads to reliable results (the trend of Nusselt number was converged) where a comparison between 663,000 with 800,000 elements showed that the local Nusselt number's deviation reached 0.63%. Therefore, considering the precision and computational cost, element number 663000 was considered to model the present work.

### Model validation

**Figure 3:**
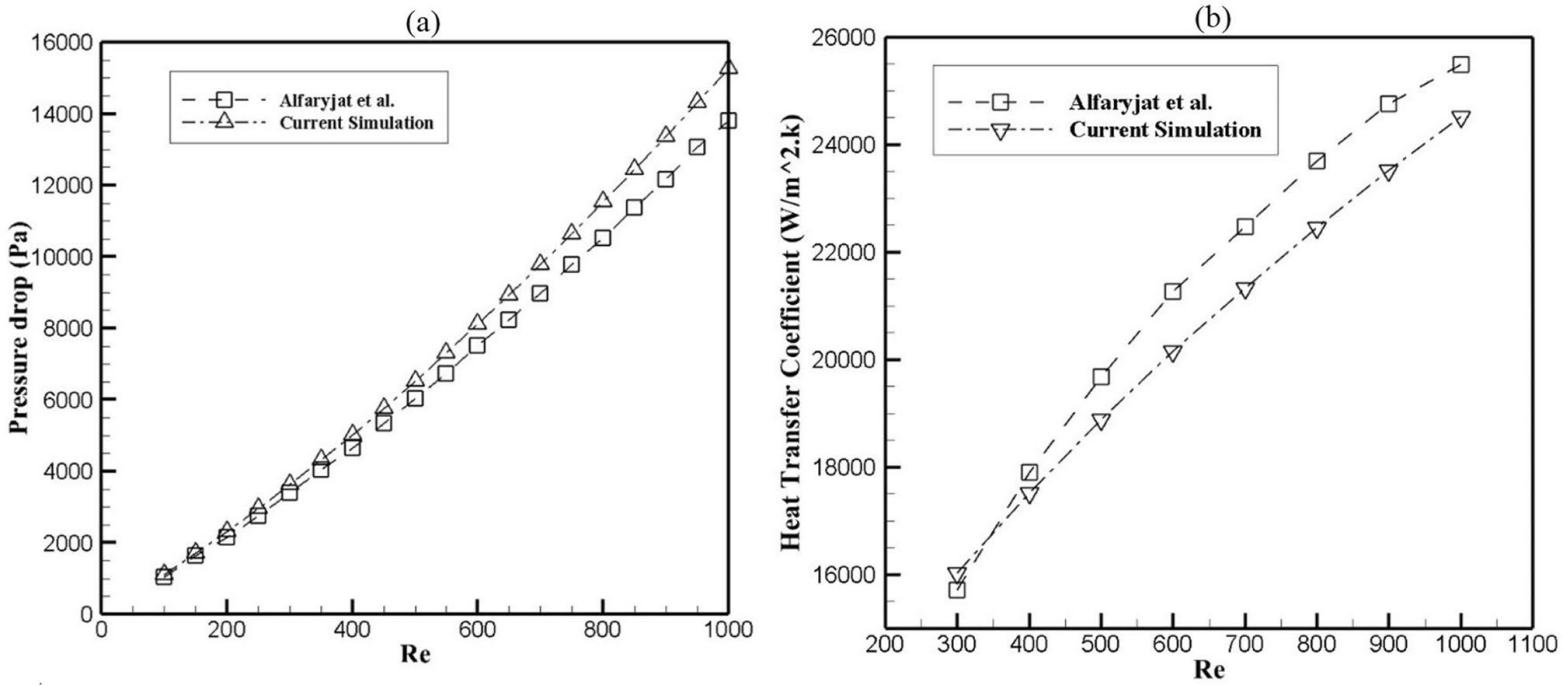
Validation of (**a**) Pressure drop and (**b**) Heat transfer coefficient at different Reynolds numbers with Alfaryjat et al.^22^.

Our considered model was validated by analyzing the results of Alfaryjat et al.^22^. The pressure drop variation and the average heat transfer coefficient versus Reynolds number are calculated (see Fig. 3), and the achieved results showed a good agreement between both works. The maximum deviation of heat transfer and pressure drop occurs at the Reynolds number of 1000, equal to approximately 4% and 10%, respectively, which these deviations are acceptable as per the previous studies^29^.

## Results and discussion

### Effect of cross-section on heat transfer

**Figure 4:**
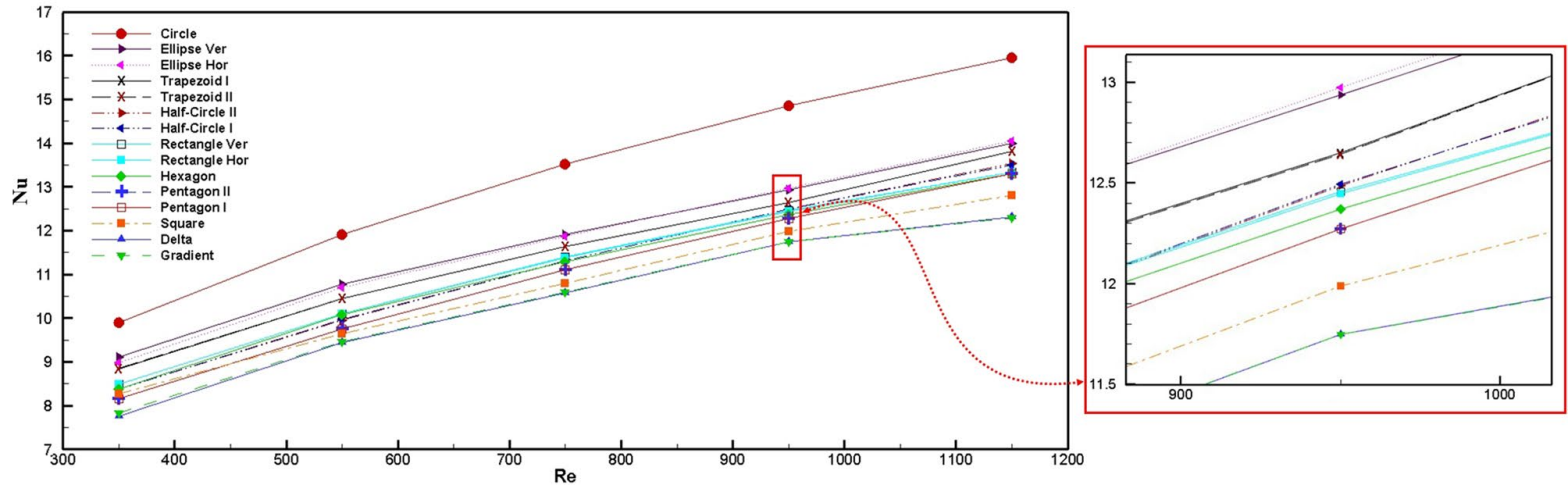
Average Nusselt number for different cross-sections of microchannel at different Reynolds numbers.

The average local Nusselt number at various Reynolds numbers for all cross-section shapes is presented in Fig. 4. The increasing Ab/NAi ratio (wetted area) in the heat transfer equations enhances the heat transfer coefficient and the Nusselt number, and according to Table 1, trapezoid and circle have the highest Ab/NAi compare to other shapes. On the other hand, the temperature differences between the liquid and the solid in the microchannel heat sink can increase the Nusselt number, in which the minimum temperature difference belongs to the circle, which was obtained from simulation results. As all of the cross-sections have an equal hydraulic diameter, the wetted area is the parameter that directly affects heat transfer enhancement. The bigger the wetted area, the more enhancement in heat transfer occurs. The wetted area results are presented in Table 2. In addition, when two cross-sections have approximately equal wetted areas, the hotspot zone as another effective parameter can influence heat transfer and overcome the wetted area parameter. As shown in Fig. 6, hot spot zones, for instance, in the trapezoid, produce a maldistribution in temperature and prevent the heat from being fully and appropriately transferred through the microchannel. Hence, in cross-sections that have corners, these zones have adverse effects on heat transfer. Due to the abovementioned reasons, the circle has the highest Nusselt number, and the shape of the gradient has the lowest value, as is shown in Fig. 4. Besides, the orientation of cross-sections for ellipse, trapezoid, and triangle can lead to Nusselt number enhancement, while the effect of this parameter on the Nusselt number for rectangle, half-circle, and pentagon shapes can be neglected.

### Investigation of fluid flow pattern

**Figure 5:**
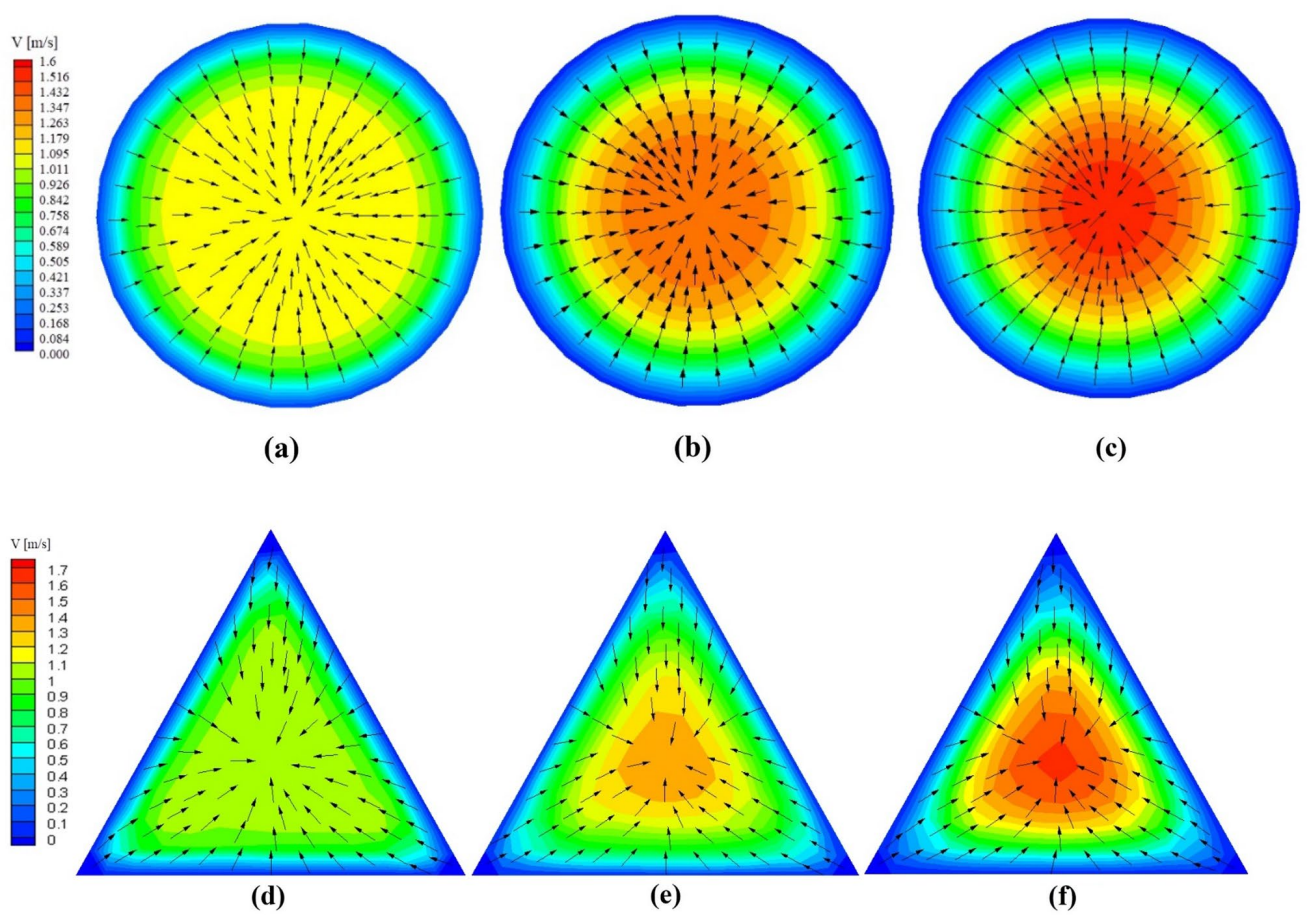
Velocity contours and vectors of circular cross-section at (**a**) x′ = 0.1, (**b**) x′ = 0.5, (**c**) x′ = 0.9, and triangular cross-section at (**d**) x′ = 0.1, (**e**) x′ = 0.5, (**f**) x′ = 0.9.

**Figure 6:**
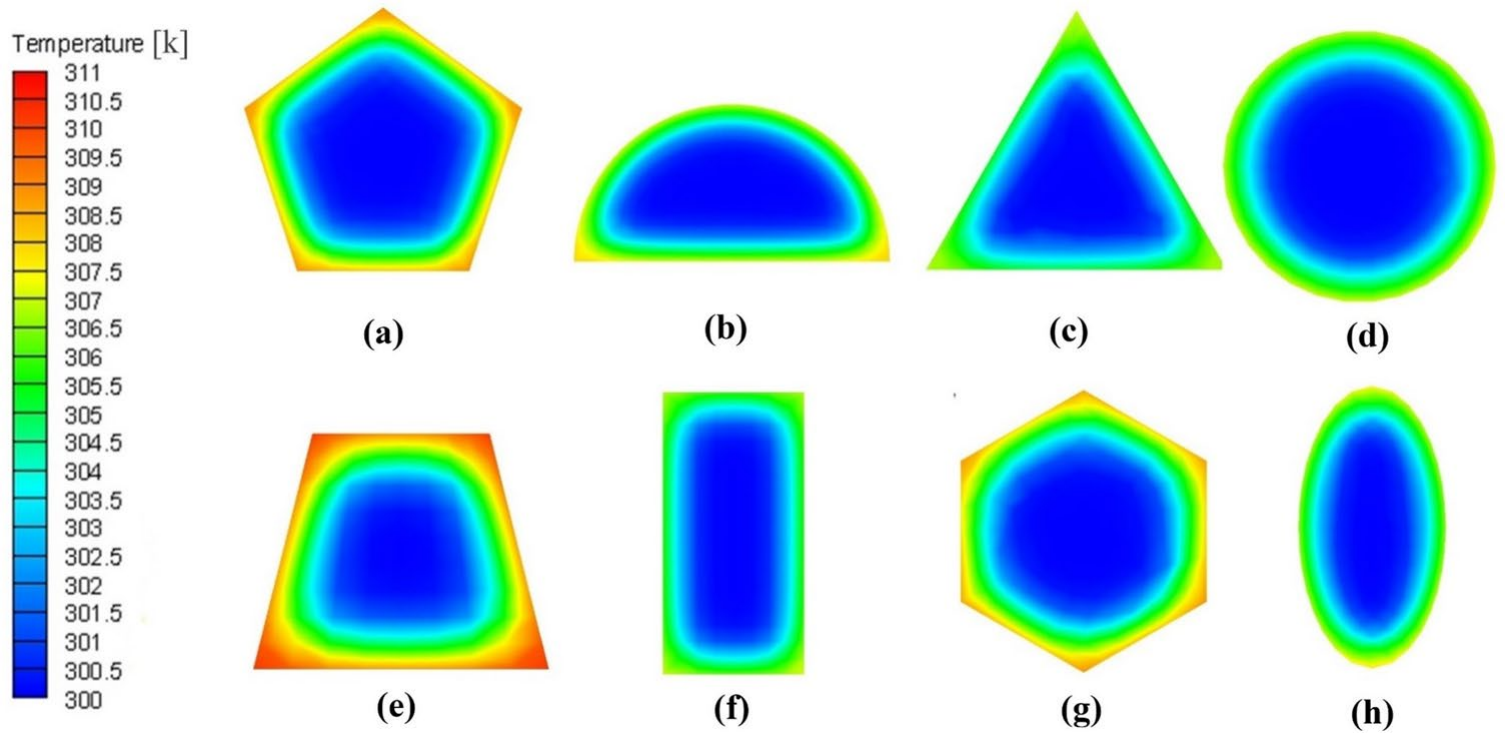
Temperature distribution contours for eight of the selected distinguished cross-sections.

According to Fig. 5, the velocity of the cross-sections is increased along the microchannel. Also, Fig. 5 shows that velocity distribution (at Re = 550) in the circle is more uniform than other cross-sections (see Section 1 of SI), which are caused to decrease velocity maldistribution and increase the heat transfer. The dead zones or places with zero velocity usually occurred at the cross-section's sharp angles, so the ehnacement of temperature at these points is observed. Besides, in the delta and gradient shapes, velocity maldistribution is increased and leads to dead zones at the angles, which is caused to generate hotspots that decrease heat transfer rate (Fig. 6). Fig. S1 (Section 1 of SI) shows velocity contour and vectors of the circle at Reynolds numbers of 350, 750, and 1050. As per Fig. 6, the more increase in Reynolds number, the more increase in the maximum velocity of cross-section, so the Nusselt number is increased at high Reynolds number.

### Temperature distributions

One of the critical parameters of thermal performance is the temperature distribution in the working fluid by which the points with high temperatures can be determined. High-temperature points can reduce the efficiency of the thermal heat sink in the microchannel by creating thermal maldistribution. Figure 6 shows the temperature distribution of the eight cross-sections at the microchannel outlet at Reynolds number 550. According to Fig. 6, trapezoidal, hexagonal, and pentagonal shapes had hotspot points in their edges; for this reason, the temperature maldistributions are increased compared to other edgeless cross-sections. Consequently, the decrement of Nusselt numbers of mentioned cross-sections presented in Fig. 4 is approved by temperature distribution contours (Fig. 6).

### Pressure drop

**Figure 7:**
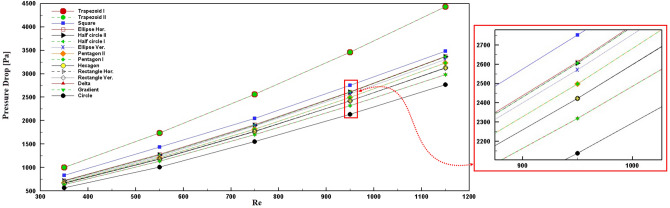
Pressure drops for different cross-sections of microchannel at different Reynolds numbers.

The pressure drop plays a critical role in the cooling system's efficiency because this parameter determines the type and the pumping pressure that affects pumping power. An increment in pressure drop leads to higher pumping power usage for electronic cooling, which causes efficiency decrement. According to Fig. 7, the trapezoid shape creates the highest pressure drop during the cooling, while the circle cross-section pressure drop is the lowest value compared with other shapes. Other cross-section pressure drops are almost close to each other, so this penalty could be compensated by selecting a suitable pump. Besides, the shape orientation does not affect the pressure drop.

### Performance evaluation criteria index of cross-sections

To evaluate the microchannel heat sink's overall performance, increasing the heat transfer rate and the pressure drop must be considered. The weight of these increases can be determined by the performance evaluation criteria index (ƞ). Since horizontal rectangular is the typical cross-section used in the commercial samples, other shapes were normalized to rectangular. When in one case, ƞ is higher than 1, it means that the overall performance of this case is higher than the rectangular cross-section.

**Figure 8:**
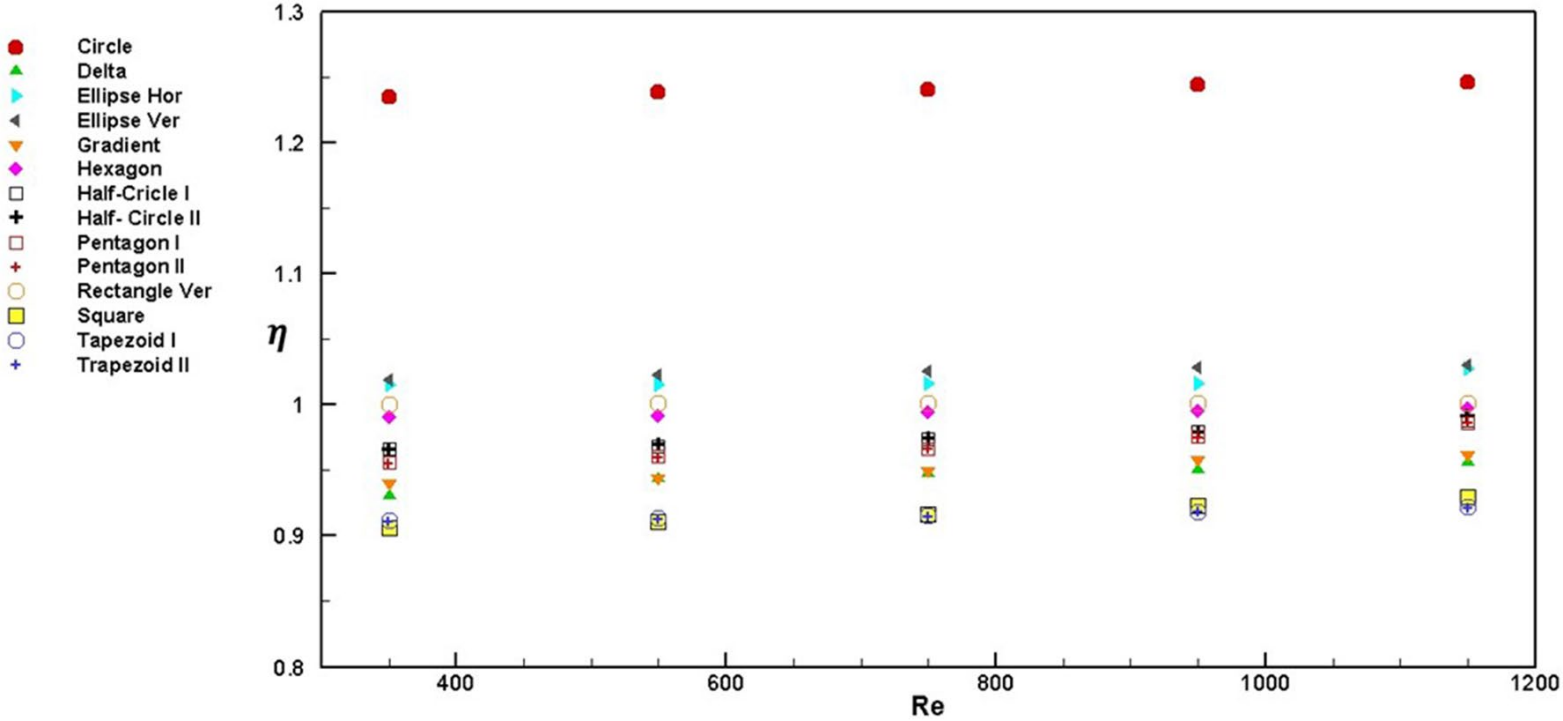
Performance evaluation criteria index of different cross-sections at different Reynolds numbers.

Figure 8 shows ƞ at different Reynolds numbers for all studied cross-sections. The circle and trapezoid geometries present maximum and minimum ƞ value, respectively; see Fig. 8. It means increasing the heat transfer preponderates to the pressure drop penalty in the circle cross-section^24,30–32^.

11 $$E_{Nu} = \frac{{Nu_{Cross - section} }}{{Nu_{Rectangle} }}$$

12 $$E_{\Delta P} = \frac{{\Delta P_{Cross - section} }}{{\Delta P_{Rectangle} }}$$

13 $$\eta = \frac{{\frac{{Nu_{Cross - section} }}{{Nu_{Rectangle} }}}}{{\left( {\frac{{\Delta P_{Cross - section} }}{{\Delta P_{Rectangle} }}} \right)^{\frac{1}{3}} }} = \frac{{\left( {E_{Nu} } \right)}}{{\left( {E_{\Delta P} } \right)^{\frac{1}{3}} }}$$

### Liquid cooling application in PCR

Approximately all PCR devices include three main steps^33,34^, and Table 4 shows the entire DNA amplification procedure.

**Table 4:** Steps of the standard PCR devices.

Repetition	1 X	35 X			1 X
Steps	1	2	3	4	Maintenance
Temp (℃)	94	94	54	72	Keep at 4
Hold for (s)	120	40	40	60	∞

At the beginning (steps 1 & 2), the samples must reach and be held at 94 ℃ for 160 s as the denaturation step, followed by decreasing temperature to 54 ℃ for 40 s for annealing. To elongate DNAs, the temperature must rise to 72 ℃ and be held for 60 s after the termination of the last step (step 4). The amplification cycle is started and steps 2–4 are repeated 35 times to complete the process. Finally, in order to keep the samples, they should be kept at 4 °C. The importance of cooling is significant, especially from steps 2 to step 3. The conventional cooling methods are used to apply air cooling to decrease the temperature. Mulberry et al.^35^ calculated the time in real-time PCR aid by the fan and reported 20.97 s as the cooling time for each cycle.

Since the thermal conductivity of air is lower than liquids, water cooling is used to improve the heat transfer rate in the current work. Therefore, the microchannel heat sink is implemented at the bottom of the PCR device.

According to the simulations implemented and indicated in previous sections, the circle cross-section has the most heat transfer efficiency. Thus, this geometry is used as the cross-section of the microchannel heat sink installed at the bottom of the PCR plate. The mesh independent geometry of the microchannel obtained in the previous section is used in this simulation. For simplifying the study, instead of studying the whole structure (geometry of 96 wells PCR plate structures as shown in Fig. 1a), a single structure of PCR device and PCR plate with a microchannel attached to the bottom surface of the apparatus (Fig. 1b) were investigated numerically, with four side faces of the geometry considered symmetry. The dimensions of this structure are 32.25 × 8 × 8.26 mm (as height, width, and length of the device, respectively).

**Figure 9:**
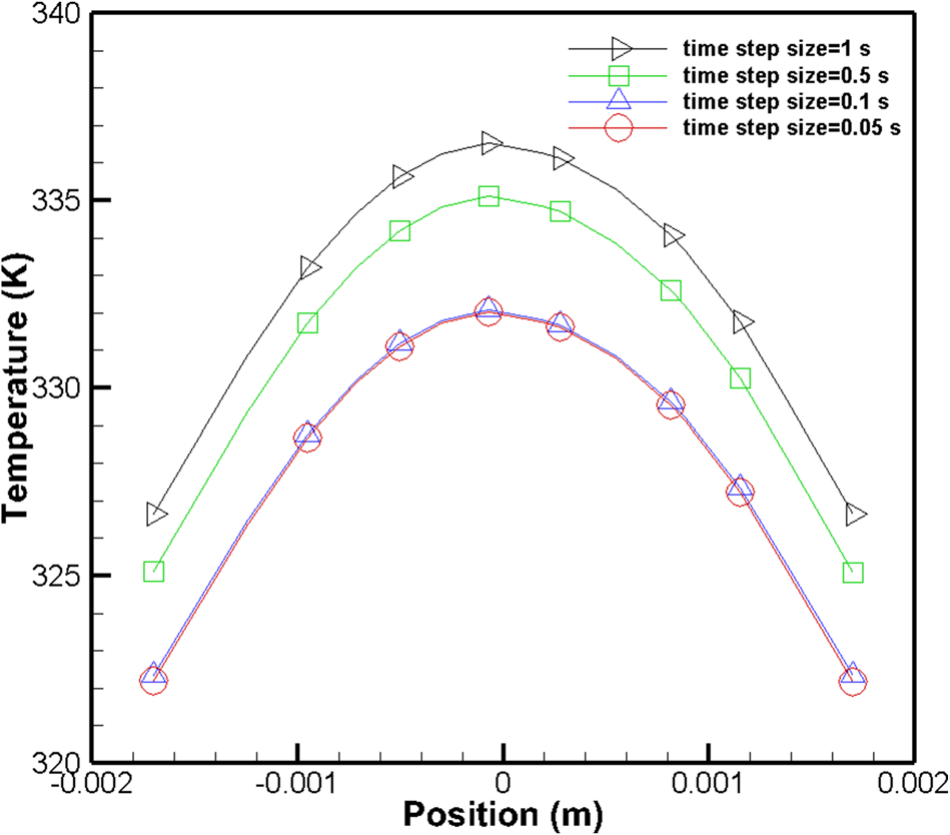
Time step validation with four different time step sizes at the center of PCR device.

In order to attain the amount of time needed to cool the sample, a transient simulation is studied by ANSYS Fluent ver. 18.2 software. For obtaining time steps independent results, four different time step sizes versus temperature were analyzed at the center of the PCR device, which can be seen in Fig. 9. hence, a time step size of 0.1(s) was selected for the simulation. The sinusoidal procedure of heating and cooling the saliva sample were applied to the software, according to Table 4 by adding a transient table (refer to Section 2 of SI) to the simulation in ANSYS for the heater element surface during the process (refer to the movies in the SI). The investigation revealed that it takes 16.5 s in each cycle to cool the samples of DNA to the required temperature, which is significant compared to air-cooling. The time difference between air and water cooling is approximately 4.5 s, which adds up to 157.5 s for the whole procedure^35^. This amount of time can easily be prevented by using this state-of-art technology. Besides, this method can save considerable room. The bulky air-cooling technique needs much bigger space compared to the miniature liquid-cooling system.

## Conclusions and future directions

In the current study, 15 cross-sections of microchannel heat sink at Reynolds number range between 350 and 1150 are investigated numerically. The main findings are listed below:

- Nusselt number for all shapes is calculated, and accordingly, the circle cross-section has the most value between other shapes at all of the surveyed Reynolds numbers. For instance, at the Reynolds number of 1150, the enhancement of the Nusselt number for the circle cross-section compared to the square cross-section is approximately 25%. On the other hand, the delta cross-section has the minimum value of the Nusselt number at low Reynolds numbers. Conversely, the gradient and square cross-sections have the minimum value at higher Reynolds numbers.
- Pressure drops for all of the shapes are calculated. As a result, the trapezoid Ι and ΙΙ have the highest amount of pressure drop among the other cross-sections at every Reynolds number. In addition, the circle cross-section has the minimum value of pressure drop at each Reynolds number among the other cross-sections.
- To better understand thermal performance, the PEC index is introduced and calculated for all cross-sections. Consequently, the circle cross-section has the highest PEC value and is an efficient choice for the microchannel heat sink.
- With a selection of the optimum cross-section, the PCR cycle was simulated numerically. The findings show that the liquid cooling time is 16.5 s, which is 157.5 s difference between air cooling for the whole procedure.
- The overall geometry of the PCR device could be reduced to a 96-well PCR plate using the microchannel heat sink instead of a bulky PCR enclosure.

Finally, after investigating liquid cooling applications using microchannel heat sink in PCR as an effective combination of microfluid with micro-scale heat transfer, some research gaps made an appearance. Therefore, researchers could be focused on the following issues to reach high-quality results:

- investigating the different patterns, for instance, using oblique shapes in the pattern of microchannel heat sinks for PCR application
- Studying the effect of nanofluids as the heat transfer fluid in microchannel heat sinks for PCR application
- Studying the effect of novel hybrid nanofluids as the heat transfer fluid in microchannel heat sinks for PCR application.

## Supplementary Information

Supplementary Information 1.

Supplementary Video 1.

Supplementary Video 2.

## Abbreviations

*A* (m^2^): Area
*B* (m^2^): Base
*C*_*p*_ (J/K kg): Specific heat capacity at constant pressure
*D* (µm): Diameter
*E*_*f*_: Pressure drop penalty factor
*E*_*Nu*_: Heat transfer enhancement factor
*h*: Heat transfer coefficient
*H* (µm): Height
*k* (W/m K): Thermal conductivity
*L* (µm): Length
*l* (µm): Length of fin
*N*: Number of fin row
*Nu*: Nusselt Number
*p* (µm): Pitch
*P* (Pa): Pressure
*∆P* (Pa): Pressure drop
q″ (W/m^2^): Heat flux
*Re*: Reynolds Number
*T* (K): Temperature
*u* (m/s): X-coordinate velocity
*v* (m/s): Y-coordinate velocity
*w* (m/s): Z-coordinate velocity
*W* (µm): Width
*X**: Normalized length
*X, Y, Z*: Global coordinate system

## Greek symbol

*γ*: The ratio of the groove width to the fin length
*θ* (Degree): Angle
*µ* (kg /m s): Dynamic viscosity
*ρ* (kg/m^3^): Density
*η*: Performance evaluation criteria index

## Subscripts

*b*: Bottom surface
*c*: Cross-section
*ch*: Channel
*f*: Fluid
*h*: Hydraulic
*i*: Solid and fluid total surface
*in*: Inlet
*l*: Average
*o*: Oblique fin
*out*: Outlet
*s*: Solid
*x*: Local

## References

[1] Rahman MT, Uddin MS, Sultana R, Moue A, Setu M (2013). Polymerase chain reaction (PCR): A short review. Anwer Khan Mod. Med. Coll. J..

[2] Mullis KB (1994). The polymerase chain reaction (Nobel Lecture). Angew. Chemie Int. Ed. English.

[3] Mullis KB (1990). The unusual origin of the polymerase chain reaction. Sci. Am..

[4] Cha RS, Thilly WG (1993). Specificity, efficiency, and fidelity of PCR. PCR Methods Appl..

[5] Khater A, Abdelrehim O, Mohammadi M, Mohamad A, Sanati-Nezhad A (2021). Thermal droplet microfluidics: From biology to cooling technology. TrAC Trends Anal. Chem..

[6] Khan WA, Yovanovich MM, Culham JR (2006). Optimization of microchannel heat sinks using entropy generation minimization method. Annu. IEEE Semicond. Therm. Meas. Manag. Symp..

[7] Kleiner MB, Kuehn SA, Haberger K (1995). High performance forced air cooling scheme employing microchannel heat exchangers. IEEE Trans. Comp. Packag. Manuf. Technol. Part A.

[8] Dix, J. & Jokar, A. A microchannel heat exchanger for electronics cooling applications Joseph. *Sixth Int. ASME Conf. Nanochannels Microchannels Minichannels ICNMM2008* 2 (2018).

[9] Baghernezhad, N. & Abouali, O. ICNMM2008-–62262. 1–8 (2016).

[10] Alihosseini Y, Zabetian Targhi M, Mahdi Heyhat M (2021). Thermo-hydraulic performance of wavy microchannel heat sink with oblique grooved finned. Appl. Therm. Eng..

[11] Parlak Z (2018). Optimal design of wavy microchannel and comparison of heat transfer characteristics with zigzag and straight geometries. Heat Mass Transf. und Stoffuebertragung.

[12] Bowers J (2018). Flow and heat transfer behaviour of nanofluids in microchannels. Prog. Nat. Sci. Mater. Int..

[13] Liu D, Lee PS, Garimella SV (2005). Prediction of the onset of nucleate boiling in microchannel flow. Int. J. Heat Mass Transf..

[14] Hasan MI, Tbena HL (2018). Using of phase change materials to enhance the thermal performance of micro channel heat sink. Eng. Sci. Technol. Int. J..

[15] Zhao N, Guo L, Qi C, Chen T, Cui X (2019). Experimental study on thermo-hydraulic performance of nanofluids in CPU heat sink with rectangular grooves and cylindrical bugles based on exergy efficiency. Energy Convers. Manag..

[16] Zhao N, Qi C, Chen T, Tang J, Cui X (2019). Experimental study on influences of cylindrical grooves on thermal efficiency, exergy efficiency and entropy generation of CPU cooled by nanofluids. Int. J. Heat Mass Transf..

[17] Qi C, Tang J, Fan F, Yan Y (2020). Effects of magnetic field on thermo-hydraulic behaviors of magnetic nanofluids in CPU cooling system. Appl. Therm. Eng..

[18] Deng D, Pi G, Zhang W, Wang P, Fu T (2019). Numerical study of double-layered microchannel heat sinks with different cross-sectional shapes. Entropy.

[19] Wang H, Chen Z, Gao J (2016). Influence of geometric parameters on flow and heat transfer performance of micro-channel heat sinks. Appl. Therm. Eng..

[20] Gunnasegaran P, Mohammed HA, Shuaib NH, Saidur R (2010). The effect of geometrical parameters on heat transfer characteristics of microchannels heat sink with different shapes. Int. Commun. Heat Mass Transf..

[21] Tullius JF, Tullius TK, Bayazitoglu Y (2012). Optimization of short micro pin fins in minichannels. Int. J. Heat Mass Transf..

[22] Alfaryjat AA, Mohammed HA, Adam NM, Ariffin MKA, Najafabadi MI (2014). Influence of geometrical parameters of hexagonal, circular, and rhombus microchannel heat sinks on the thermohydraulic characteristics. Int. Commun. Heat Mass Transf..

[23] Salimpour MR, Sharifhasan M, Shirani E (2011). Constructal optimization of the geometry of an array of micro-channels. Int. Commun. Heat Mass Transf..

[24] Alihosseini Y, Zabetian Targhi M, Heyhat MM, Ghorbani N (2020). Effect of a micro heat sink geometric design on thermo-hydraulic performance: A review. Appl. Therm. Eng..

[25] Alihosseini Y, Bari AR, Mohammadi M (2021). Effective parameters on increasing efficiency of microscale heat sinks and application of liquid cooling in real life. Adv. Microfluid. Nanofluids.

[26] Pollock DD (2018). Physical properties of materials for engineers. Phys. Prop. Mater. Eng..

[27] White FM (2007). Fluid mechanics McGraw-Hill series in mechanical engineering. Univ. Rhode Isl..

[28] Lin L, Zhao J, Lu G, Wang XD, Yan WM (2017). Heat transfer enhancement in microchannel heat sink by wavy channel with changing wavelength/amplitude. Int. J. Therm. Sci..

[29] Lee YJ, Singh PK, Lee PS (2015). Fluid flow and heat transfer investigations on enhanced microchannel heat sink using oblique fins with parametric study. Int. J. Heat Mass Transf..

[30] Khanesheshdar, S., Zeinali Heris, S., Shokrgozar, M. & Kahani, M. Pressure drop and thermal performance of CuO/ethylene glycol (60%)- Water (40%) nanofluid in car radiator. *Korean J. Chem. Eng.* **32** (2015).

[31] Webb RL (1981). Performance evaluation criteria for use of enhanced heat transfer surfaces in heat exchanger design. Int. J. Heat Mass Transf..

[32] Rakhsha M, Akbaridoust F, Abbassi A, Majid SA (2015). Experimental and numerical investigations of turbulent forced convection flow of nanofluid in helical coiled tubes at constant surface temperature. Powder Technol..

[33] Li Q, Zhou X (2009). Sensitive detection and quantitation of EZH2 expression in cancer cell by an electrochemiluminescent method. Eight Int. Conf. Photonics Imaging Biol. Med. (PIBM 2009).

[34] deWaard JR, Ivanova NV, Hajibabaei M, Hebert PDN (2008). Assembling DNA barcodes. Analytical protocols. Methods Mol. Biol..

[35] Mulberry G, White KA, Vaidya M, Sugaya K, Kim BN (2017). 3D printing and milling a real-time PCR device for infectious disease diagnostics. PLoS ONE.

