# Supplementary material for: Effect of liquid cooling on PCR performance with the parametric study of cross-section shapes of microchannels
Source: Sci Rep. 2021 Aug 9;11:16072. doi: 10.1038/s41598-021-95446-0 (PMC8352922; doi:10.1038/s41598-021-95446-0)
Supplement: Supplementary file 1 — Supplementary Information 1. [file 41598_2021_95446_MOESM1_ESM.docx]

**Effect of liquid cooling on PCR performance with the parametric study of cross-section shapes of microchannels**

Yousef Alihosseini ^1^**^†^**, Mohammad Reza Azaddel ^1†^, Sahel Moslemi^2^, Mahdi Mohammadi ^3,4^* Ali Pormohammad^3^, Mohammad Zabetian Targhi ^1*^, Mohammad Mahdi Heyhat ^1^

1. Faculty of Mechanical Engineering, Tarbiat Modares University, Tehran, Iran
2. Faculty of Chemical Engineering, Sahand University of Technology, Tabriz, Iran
3. Department of Mechanical and Manufacturing Engineering, University of Calgary, Calgary, Alberta T2N 1N4,
4. Biological Science Department, University of Calgary, Calgary, Alberta T2N 1N4, Canada

**Supplementary Information**

In this document, the supplementary figures and table of the simulations are presented.

**Section 1. Supplementary velocity figures**


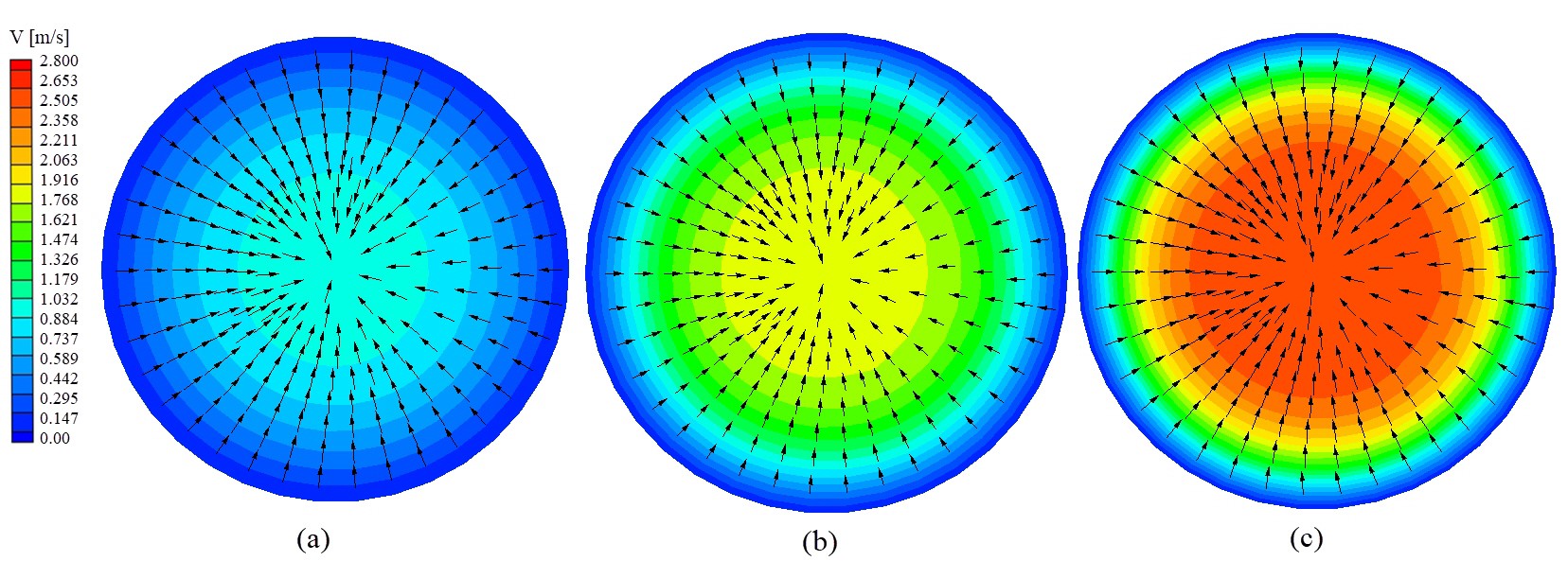
Velocity contours and vectors of circle cross-section at three Reynolds numbers are presented in Fig. S. 1 (a) Re= 350, (b) Re= 750, and (c) Re= 1050. With the increment of Reynolds number, the velocity is increased at the cross-section of channels. According to the Fig. S.1, the velocity near the walls are zero. Thus, the no-slip condition is met at the microchannel walls. The velocity profiles demonstrate that the stream lines converged at the center of the channel. Therefore, the center of the microchannels, have the maximum velocity magnitude.

Fig. S. 1 Velocity contours and vectors of circle cross-section at (a) Re= 350, (b) Re= 750, and (c) Re= 1050

Velocity contours and vectors of 6 differnet cross-sections at (a) inlet, (b) middle, and (c) outlet of the microchannels are presented in Figures S. 2 - S. 7 for further assessments. The developing region has more heat transfer rate than the developed region. For this reason, researchers always seek methods to maintain the flow in the developing region. Besides, the entrance length plays a significant role in the heat transfer. There are many ways to keep flow in the developing region such as changing the patterns and cross-section shapes or adding fins. The entrance length is calculated by l_d_ = 0.06 × Re × D_h_. Hence, the minimum entrance lengths occurs in the lowest Reynolds number which is equal to 11.1 mm at Re = 350. In the current study, the length of the simulated microchannel is 8.26 mm; consequently, the investigated region maintains in the developing state.


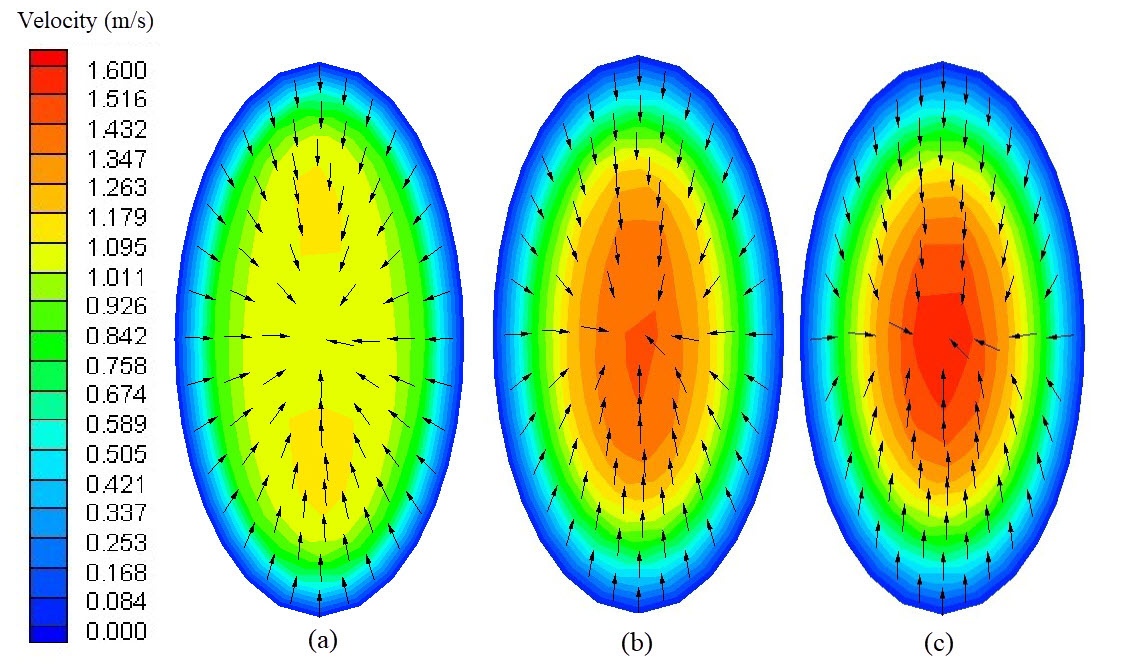


Fig. S. 2 Velocity contours and vectors of ellipse cross-section at (a) inlet, (b) middle, and (c) outlet of the microchannels at Re=550


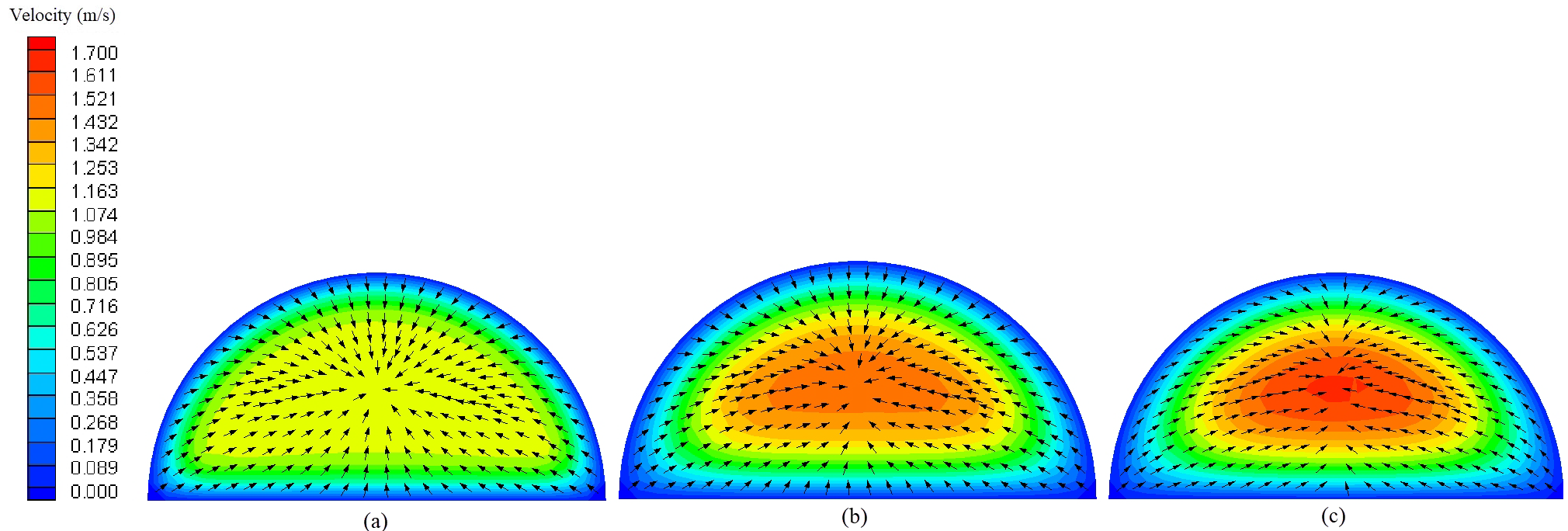


Fig. S. 3 Velocity contours and vectors of half circle cross-section at (a) inlet, (b) middle, and (c) outlet of the microchannels at Re=550


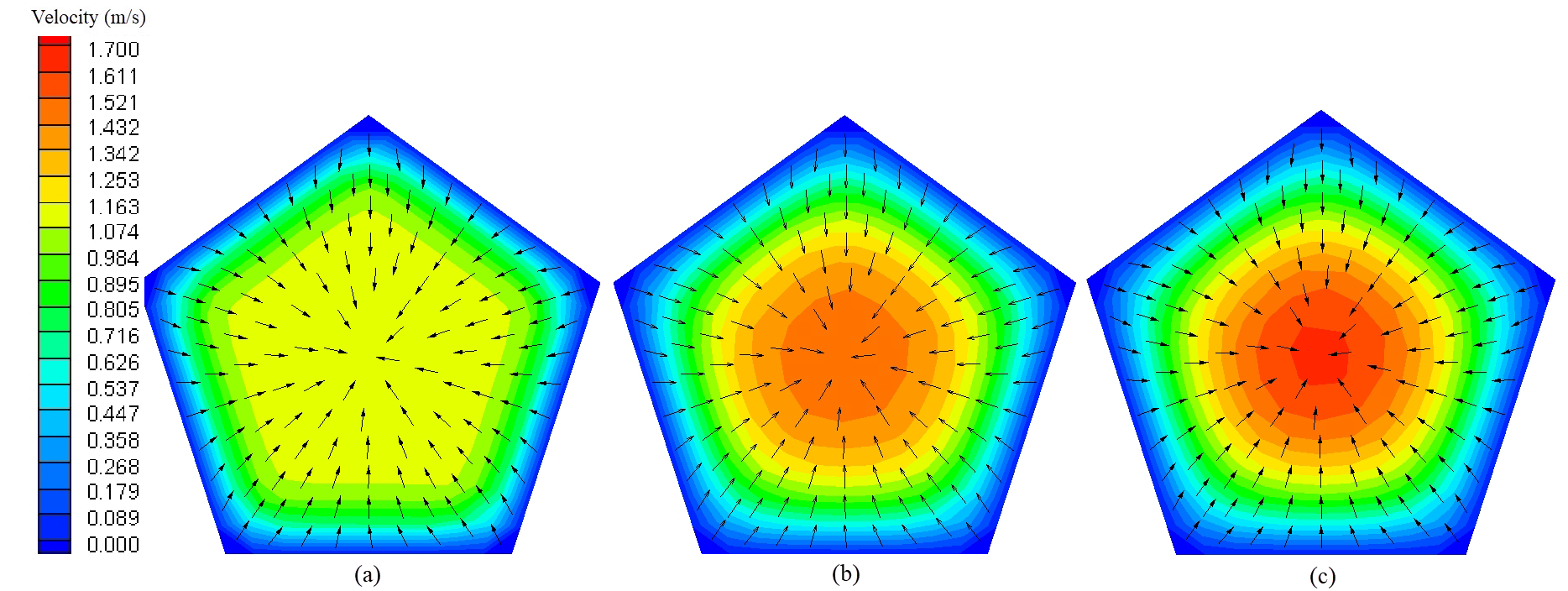


Fig. S. 4 Velocity contours and vectors of pentagon cross-section at (a) inlet, (b) middle, and (c) outlet of the microchannels at Re=550


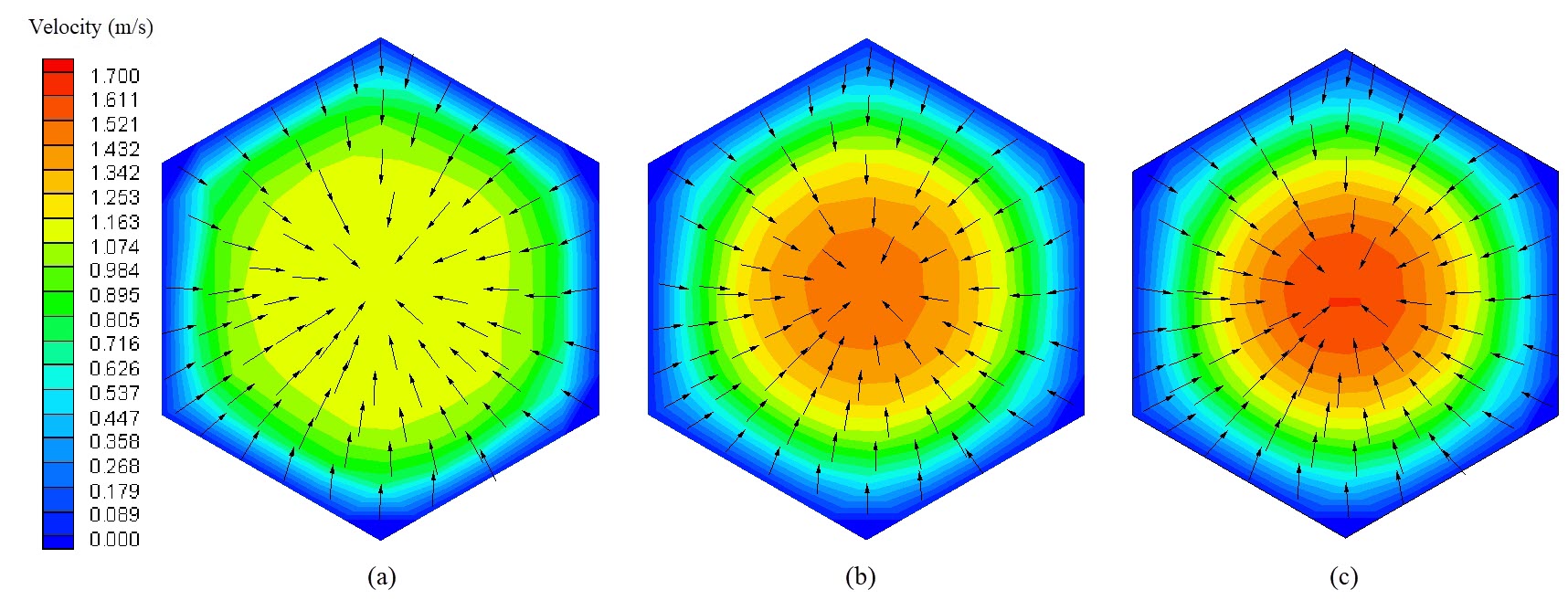


Fig. S. 5 Velocity contours and vectors of hexagon cross-section at (a) inlet, (b) middle, and (c) outlet of the microchannels at Re=550


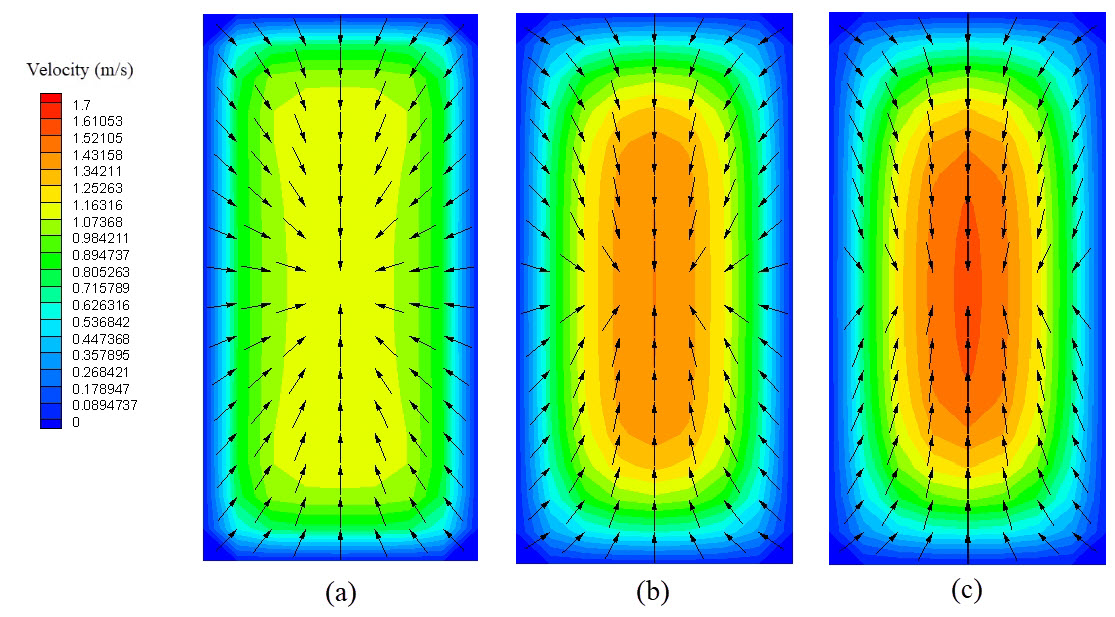


Fig. S. 6 Velocity contours and vectors of rectangle cross-section at (a) inlet, (b) middle, and (c) outlet of the microchannels at Re=550


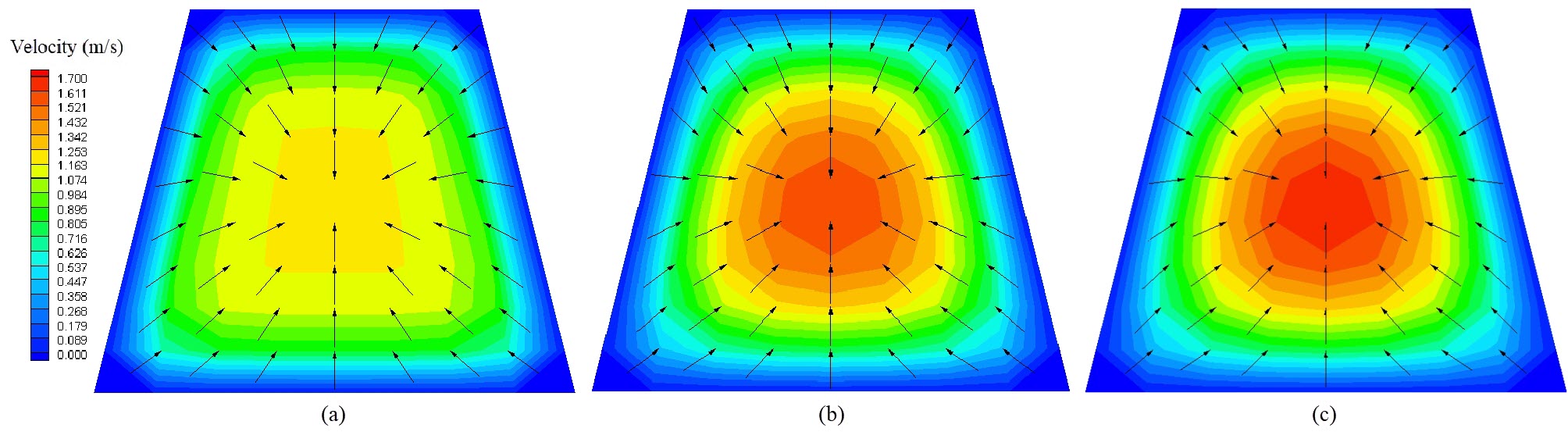


Fig. S. 7 Velocity contours and vectors of trapezoid cross-section at (a) inlet, (b) middle, and (c) outlet of the microchannels at Re=550

**Section 2. The simulation procedure of PCR cooling**

After selecting the circle channel as the most efficient cross-section, the physics of the PCR device was designed in SOLIDWORKS. Then, the geometry was exported into design modeler in ANSYS FLUENT ver. 18.2. The various sections of the PCR device such as inlet, outlet, heater, and PCR well and walls were defined and named respectively. Moreover, the geometry was meshed in ANSYS MESHING by using edge and body sizing. Since this issue was divided into two steps including heating and cooling steps (steps 1 and 2 respectively); Consequently the procedure of the simulation was divided into two parts as well. Also, the setup was set by following below steps:

1. General:
   1. Solver: Pressure-based type; Transient modeling
2. Models:
   1. Energy: on
3. Materials:
   1. Fluids: saliva sample; water-liquid
   2. Solids: Copper; Plastic
4. Boundary conditions:
   1. Inlet: the velocity of inlets were modified by inlet Reynolds number.
      1. Step 1: 0
      2. Step 2: 0.884925 m/s
   2. Outlet: were considered as outflow.
   3. Lateral walls: Isolated.
   4. Heater:
      1. Step 1: 57130 W/m­^2^
      2. Step 2: considered as a coupled wall to provide conduction heat transfer
5. Solution method:
   1. SIMPLE was chosen as the pressure-velocity coupling method.
   2. Spatial descretization of momentum, energy, and pressure were second order upwind.

**Section 3. Supplementary Table**

Transient table for designating the heatflux and velocity boundary condition in FLUENT software is presented as below. the implemented heat flux value is 57130 (55% of the nominal capacity of the heater) due to losses in the heater.

Table. S. 1 heater boundary condition transient table

| Time step | heat flux (W/m^2^) | Velocity (m/s) |
| --- | --- | --- |
| 1-510 | 57130 | 0 |
| 511-800 | 0 | 0 |
| 801-970 | 0 | 0.884925 |
